# Acute Hemorrhagic Conjunctivitis caused by Coxsackievirus A24 Variant, South Korea, 2002

**DOI:** 10.3201/eid0908.030190

**Published:** 2003-08

**Authors:** Myoung-don Oh, Sangwon Park, Youngju Choi, Hongbin Kim, Kiduk Lee, Wanbum Park, Youngae Yoo, Eui-Chong Kim, Kangwon Choe

**Affiliations:** * Seoul National University College of Medicine, Seoul, Republic of Korea; † Hamchun Eye Clinic, Seoul, Republic of Korea; ‡ Seoul National University Hospital, Seoul, Republic of Korea

**Keywords:** acute hemorrhagic conjunctivitis, coxsackievirus A24 variant, South Korea, dispatch

## Abstract

In summer 2002, a nationwide outbreak of acute hemorrhagic conjunctivitis occurred in South Korea. The etiologic agent was confirmed as coxsackievirus A24 variant (CA24v) by virus isolation and sequencing of a part of the VP1 gene. Phylogentic analysis, based on the protease 3C sequences, showed that the Korean isolates were clustered into a lineage distinct from the CA24v isolates reported in previous outbreaks in Asia.

Acute hemorrhagic conjunctivitis (AHC) is characterized by sudden onset of painful, swollen, red eyes with subconjunctival hemorrhages and excessive tearing. Most cases are self-limited but highly contagious, with the potential for causing considerable illness. Adenoviruses and picornaviruses can cause AHC outbreaks (*1*). Among picornaviruses, enterovirus 70 and coxsackievirus A24 variant (CA24v) have caused large outbreaks of AHC (*2*).

During the summer of 2002, a nationwide outbreak of AHC occurred in South Korea, which affected more than one million people. The epidemic started in late August and reached its peak in mid-September, when 1,100 schools were closed. The epidemic ended in early October. We report the investigation into the viral etiology of this outbreak.

## The Study

Conjunctival swabs were taken from AHC patients at a community-based ophthalmology clinic in Seoul on September 3–16, 2002. In the initial stages of the epidemic, attempts were made to determine whether the causative agent belonged to the adenovirus or picornavirus family. To attempt to answer this question, we performed a polymerase chain reaction (PCR) assay on conjunctival specimens without pretreatment.

Conjunctival specimens from 10 AHC patients were subjected to nested PCR that used primers to common adenoviruses (AD1F, AD2F, AD2R, and AD1R) (*3*). The specimens were also subjected to reverse transcription (RT)–PCR with primers common to enteroviruses (EV1, EV2) (*4*). Of the 10 specimens, 2 yielded a 114-bp amplification product, consistent with enteroviruses. To determine if this amplification product belonged to enterovirus 70 or CA24v, PCR was performed with primers specific to each virus (*5*), which yielded a 171-bp amplification product, consistent with CA 24v. From these preliminary results, we tried to isolate the virus.

The conjunctival specimens were added into 24-well culture plates, containing HeLa cells, and incubated at 37°C. The cells were observed daily for cytopathic effects and were harvested when the cytopathic effects involved >80% of the cells. To confirm that the isolates belonged to picornaviruses, RT-PCR was carried out with PCR primers that would anneal at conserved sites in the 5′ nontranslated region of all the enteroviruses (including coxsackieviruses) (*4*). The serotypes of the isolates were determined by partial sequencing of the VP1 region, as described previously (*4*). In brief, viral RNA was extracted from an infected cell culture supernatant, by using the QIAamp Viral RNA kit (QIAGEN, Inc., Valencia, CA). The VP1 region was amplified by RT-PCR with the 188/222 primer pairs, and the nucleotide sequence was determined by the cycle-sequencing and dye terminator methods, using an automated DNA sequencer (MJ Research Gradient Cycler, Applied Biosystems, Inc., Foster City, CA). The serotype was determined by comparing the sequence of the VP1 amplicon with those contained within the database of human enterovirus and picornavirus sequences available in GenBank.

For the molecular epidemiologic study, the protease 3C region of CA24v was amplified by PCR with the D1/D2 primer pairs and sequenced as described previously (*6*). The nucleotide sequences of this region of the 14 Korean isolates from this study were compared with those of the strains reported from the Asian countries where previous outbreaks had occurred (*6*–*8*). A phylogenetic tree was constructed by the unweighted pairwise grouping method of the arithmetic average.

Conjunctival swabs were taken from 88 AHC patients. Clinical features of the patients were consistent with the findings reported from previous outbreaks. All the patients recovered without sequelae.

Of the 88 specimens added into the HeLa cell culture, 39 (44%) showed extensive cytopathic effect on the HeLa cells. The viral RNA was extracted from the infected cell culture supernatants, and the VP1-specific fragment was amplified by RT-PCR with the 188/222 primer pairs. All 39 isolates were positive for the VP1 fragment, consistent with enterovirus. Nine of the 39 isolates were randomly selected for PCR, performed with the S3/AS3 primer pairs, which are specific to CA24v. All nine isolates yielded the 171-bp amplification product, consistent with CA24v.

The nucleotide sequences of the protease 3C region among the 14 Korean isolates of CA24v were 98.1% to 100% homologous (data not shown). A phylogenetic analysis showed that the Korean isolates were closely related and clustered to a lineage distinct from those isolates reported from the previous outbreaks in other Asian countries which had been attributed to CA24v (Figure).

**Figure:**
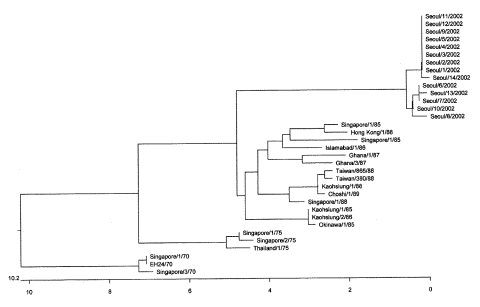
Phylogenetic analyses of the 14 isolates of coxsackievirus A24 variant (CA24v) from the South Korean outbreak of acute hemorrhagic conjunctivitis, summer 2002. The entire protease 3C region (549 nucleotides) of CA24v was amplified by polymerase chain reaction and sequenced. The nucleotide sequences of this region of the 14 Korean isolates were compared with those of the strains reported from other Asian countries in previous outbreaks. The phylogenetic tree was constructed by the unweighted pairwise grouping method of the arithmetic average. (GenBank accession nos. for the 14 Korean isolates are AY216777–AY216790.)

The 14 sequences reported here were deposited in the GenBank sequence database under the accession nos. AY216777 to AY216790. We also sequenced capsid protein VP1 region of CA24v isolates from two of our patients. GenBank accession nos. of these sequences are AF545847–AF545848.

## Conclusions

Our study demonstrated that CA24v was the causative agent of the South Korean outbreak of AHC in the summer of 2002. During this outbreak, CA24v was also isolated from patients with AHC in Busan, the second largest city in South Korea, and other provinces (*9*). During the last 3 decades, several large outbreaks of AHC have occurred in South Korea. However, in the 1970s, the causative agents of the outbreaks were rarely determined. Ishii et al. tried to isolate the virus from patients with acute conjunctivitis in Busan between April and August 1983. Of 123 patients with acute conjunctivitis, 74 (60.2%) were infected with adenoviruses (type 8 was the most prevalent, with 57 cases) and 5 (4.0%) with enterovirus 70 (*10*). Kim also reported the isolation of adenoviruses and enterovirus 70 from patients with acute conjunctivitis from Seoul between 1987 and 1990 (*11*). In 2002, CA24v was determined to be the cause of an AHC outbreak in South Korea for the first time.

An outbreak of AHC attributable to CA24v was first recognized in Singapore in 1970; outbreaks have occurred periodically there since (*12*,*13*). CA24v had not been reported outside of Southeast Asia and the Indian subcontinent until 1986, when it appeared in the Western Hemisphere in an outbreak in the Caribbean (*14*). A phylogenetic analysis, based on the proteinase 3C region of the CA24v isolates collected from the Eastern Hemisphere in 1970 through 1989, demonstrated that most of the lineages of the tree contained Singapore strains (*6*). Based on these findings, Lin et al. suggested that CA24v has been circulating endemically in Singapore and the surrounding areas and has occasionally spread to other countries (*8*). Whether CA24v was newly introduced or had already been introduced and was circulating in South Korea before the AHC outbreak in 2002 is not known. In September 2002, an outbreak of AHC was reported in southern China (*15*). However, further information on the cause of that outbreak is not available.

Although using sequences from the capsid regions of enteroviruses for molecular epidemiologic studies would have been more informative (because of discontinuities from recombination outside the capsid region), we used 3C regions for the phylogenetic analysis because sequence data for capsid regions of the CA24v isolates from previous outbreaks in Asian countries were not available (*16*). The nucleotide sequence homologies of the protease 3C region among the 14 Korean isolates of CA24v were high (98.1% to 100%), consistent with a common source outbreak. The phylogenetic analysis showed that the Korean isolates of CA24v clustered together into a distinct lineage (Figure). Our data suggests that CA24v evolved from the previous Asian isolates and caused the South Korean outbreak of AHC during the summer of 2002.
